# Investigating the Relationship Between the Expression Level of Mucin Gene Cluster (MUC2, MUC5A, and MUC5B) and Clinicopathological Characterization of Colorectal Cancer

**DOI:** 10.31661/gmj.v10i0.2030

**Published:** 2021-12-02

**Authors:** Hossein Iranmanesh, Ahmad Majd, Ehsan Nazemalhosseini Mojarad, Mohammad Reza Zali, Mehrdad Hashemi

**Affiliations:** ^1^Department of Medical laboratory, Ali Asghar Hospital, Iran University of Medical Sciences , Tehran, Iran; ^2^Department of Biology, Faculty of Sciences, North Tehran Branch, Islamic Azad University, Tehran, Iran; ^3^Gastroenterology and Liver Diseases Research Center, Research Institute for Gastroenterology and Liver Diseases, Shahid Beheshti University of Medical Sciences, Tehran, Iran; ^4^Department of Genetics, Faculty of Advanced Science and Technology, Tehran Medical Sciences, Islamic Azad University, Tehran, Iran; ^5^Farhikhtegan Medical Convergence sciences Research Center, Farhikhtegan Hospital Tehran Medical Sciences, Islamic Azad University, Tehran, Iran

**Keywords:** MUC Secretory Genes;, Colorectal Cancer;, Gene Expression

## Abstract

**Background:**

Colorectal cancer (CRC) is one of the most common cancers in the world and has a high mortality rate. It is accepted that dysfunction in the expression of mucins are associated with the occurrence and development of CRC. Therefore, the present study aimed to investigate the expression of MUC2, MUC5A, and MUC5B genes in CRC and their relationship with clinicopathological variables.

**Materials and Methods:**

The population included 28 patients after a colonoscopy and confirmation of the results. Tumors and parallel adjacent normal tissues from CRC patients were collected. RNA extraction and cDNA synthesis were performed using the corresponding kits. The gene primer was designed and RT-PCR was used to evaluate gene expression. The t-test and ANOVA were used to examine the differences between the different groups. Data analysis was performed using Prism8 software. Tumors from CRC patients were retrospectively collected from Taleghani Hospital, Shahid Beheshti University of Medical Sciences, Tehran, Iran.

**Results:**

The results showed that the expression of MUC2, MUC5A, and MUC5B genes was lower in patients with CRC aged 50 years or younger than was in older patients (P<0.05). Only the MUC5B gene expression was associated with tumor grades, which was higher in poorly differentiated tumors. The expression of MUC5A and MUC2 genes was higher in stage IV of the tumor than in other stages (P<0.05). Conclusion: Among the changes in the expression of MUC secretory genes, including MUC2, MUC5A, and MUC5B and clinicopathological variables, there was a relationship that could have prognostic and diagnostic value in CRC.

**Conclusion:**

None.

## Introduction

Colorectal cancer (CRC) is the third most common disease and the fourth deadliest cancer in the world [1]. It is the second most common cancer in women after breast cancer and the most common cancer in men after lung and prostate cancer [2]. One of the major clinical challenges of this malignancy is the late diagnosis and/or progression of the disease to metastasis. Therefore, diagnostic and predictive biomarkers are of clinical importance. Finding new biomarkers can enhance the diagnosis, differentiation, and early stage of the disease [3].>Mucins, as cell surface receptors, are involved in guiding cellular signals generated in response to external stimuli that cause proliferation, differentiation, and apoptosis in cells [4,5,6]. It has also been shown that the level of mucin secretion increases in various cancers, especially in adenocarcinoma [4,5,6]. These molecules have high molecular weights and contain tandem repeat sequences of amino acids. MUC2, MUC5A, and MUC5B are clustered on the 11p15 chromosome, in tandem, and form the secreted or gel-forming mucins [7]. MUC2 is a glycoprotein found on the surface of many epithelial cells and is naturally involved as a protector in these cells. It is also effective in differentiating epithelial cells and adhesion balance [8,9]. There are conflicting results in the literature regarding the expression of MUC2 in CRC. The results of some studies have shown that the expression of MUC2 in colorectal adenocarcinomas is reduced [1010^11^], but other studies have shown that its expression is increased [12,13]. A study also reported no change in MUC2 protein level in CRC [14]. However, animal studies have shown that the knockdown of the MUC2 gene causes the formation of tumors, first in the small intestine and then in the large intestine, due to increased cell proliferation, reduced cell death, and increased migration of intestinal epithelial cells [15]. MUC5A is another secretory MUC that is not found in the normal colon, but is expressed at the surface of the gastric epithelium and in tracheobronchial cells [16,17]. MUC5A mRNA has been identified in adenomatous polyps [18]. De novo expression of MUC5A has been detected in rectosigmoid villous adenomas using the in situ hybridization technique [19]. It has been demonstrated that MUC5A expression can be of prognostic value in many colorectal carcinomas; thus, a lack of expression of this secretory MUC can be a predictor of aggressive colorectal carcinoma [20]. Very low expression of MUC5B has been reported in colon studies [16]. MUC5B expression has been shown to be limited to colonic goblet cells [15], yet there are few studies on the association of MUC5B with CRC. Therefore, the aim of this study was to investigate the expression of MUC2, MUC5A, and MUC5B genes in normal tissues and CRC and their relationship with invasion and metastasis.

## Materials and Methods

### Patients

This study was performed on 28 patients who were referred to Taleghani Hospital, Tehran, Iran, for treatment and/or diagnosis. After a colonoscopy and confirmation of the results by a pathologist, these patients were further referred by a gastroenterologist for genetic testing in this research project. Patients were asked to answer demographic questions on a questionnaire. Thereafter, specimens were obtained individually from the patients from their seemingly healthy margins, during a colonoscopy by a gastroenterologist specializing in CRC, and placed in RNAlater solution (Sigma, Germany). The samples were immediately transferred to liquid nitrogen and stored until the RNA extraction. Exclusion criteria included patients with T1 cancer treated by endoscopic polypectomy, patients who received neoadjuvant chemotherapy, and patients with synchronous or metachronous invasive cancers originating from the colorectum or other sites [21].

### Ethical Considerations

The current study has been approved by the Ethics Committee of the Research Center for Gastroenterology and Liver Diseases, Shahid Beheshti University of Medical Sciences, Tehran, Iran (approval code IR.SBMU.RIGLD.REC.1396.180).

### RNA Extraction and cDNA Synthesis

The total RNA extraction kit (Yekta Tajhiz Azma Co., Iran) was used to extract RNA. After extraction, the quantity and quality were evaluated using Nanodrop (Thermo Fisher Scientific, NANO 300 ,UK ) and gel electrophoresis. The RevertAid RT Kit (Thermo Fisher Scientific ,UK) was used to synthesize the cDNA following manufacturer instructions for cDNA synthesis.

### Primer Design

In this study, the β-actin gene was selected as the internal control. Two pairs of primers were designed using Gene Runner software version 3.05 (Hastings Software Inc. Hastings, NY, USA, http://www. generunner.com) to design primers for MUC2, MUC5AC, and MUC5B genes. The primers were synthesized by the CinnaGen Co. (Tehran, Iran). The sequences of the primers are given in Table-1.

### Real-Time (RT) Polymerase Chain Reaction (PCR)

Relative quantitative RT-PCR was used to study the expression of MUC2, MUC5A C, and MUC5B genes. For this purpose, the Takara SYBR Premix Ex Taq II (TliRNaseH Plus,South Korea ) kit was used. Kit manufacturer instructions were followed to perform RT-PCR. Temperature conditions of RT-PCR were initial denaturation at 95°C for 10 min, with 40 cycles at 95°C for 10s, and 60°C for 30 min for annealing and extension, respectively.

### Statistical Analysis

In this study, the rate of change in the expression of the studied genes compared to the control group was investigated by method 2-ΔΔCt. Quantitative variables were expressed as mean and standard deviation (SD), and qualitative variables as frequency and percentage. The t-test was used to investigate significant differences in gene expression in tumor and healthy tissues. The statistically significant level was considered to be <0.05. GraphPad software Prism version 8(Graphpad Software Inc.,California,USA) was used to analyze the data.

## Results

**Figure 1:**
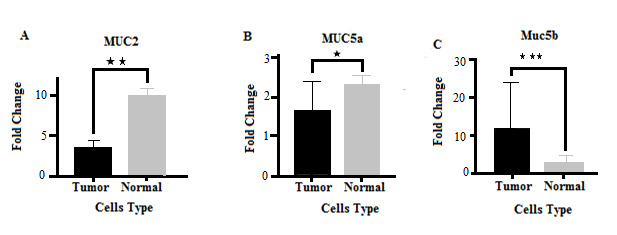
Comparison of MUC2 (**A**), MUC5A (**B**), and MUC5B (**C**) genes expression in normal and cancer cells. *: significant differences at level of P<0.05 ;*** and **: show significance differences at level of P <0.01.

**Figure 2:**
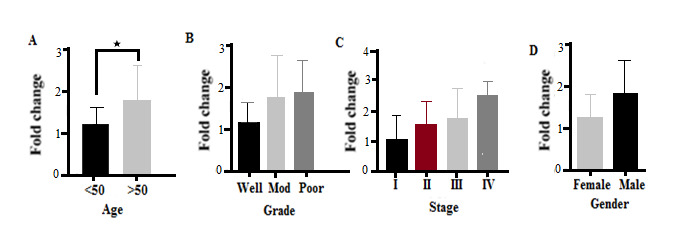
MUC5A gene expression in different patients with colorectal cancer by age (**A**), grade (**B**), stage (**C**), and gender (**D**). *: P<0.05.

**Figure 3:**
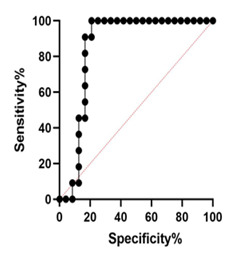
ROC curve for the potential use of MUC5A gene to identify patients with colorectal cancer

**Figure 4:**
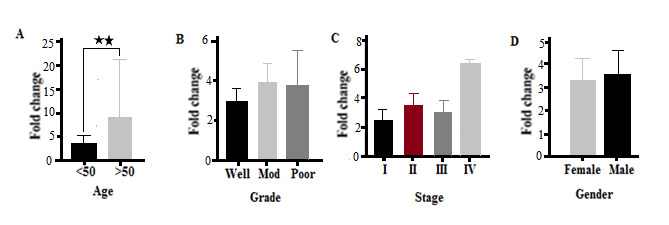
MUC2 gene expression in different patients with colorectal cancer by age (**A**), grade (**B**), stage (**C**), and gender (**D**). **: show significance differences at level of P<0.002.

**Figure 5:**
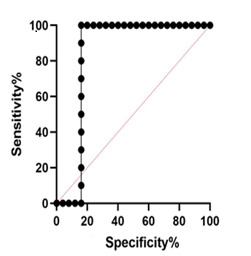
ROC curve for the potential use of the MUC2 gene to identify patients with colorectal cancer.

**Figure 6:**
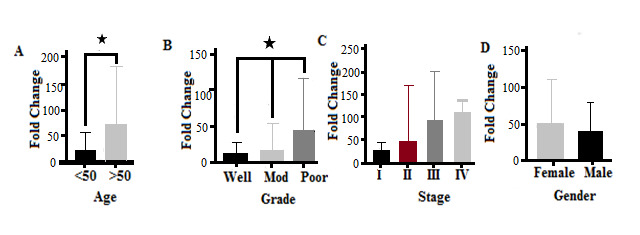
MUC5B gene expression in different patients with colorectal cancer by age (**A**), grade (**B**), stage (**C**), and gender (**D**). *: show significant differences at level of P<0.05.

**Figure 7:**
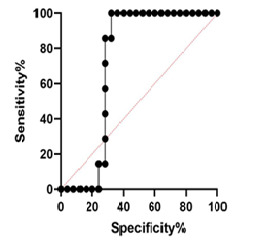
ROC curve for the potential use of MUC5B gene to identify patients with colorectal cancer.

Patient Clinicopathological Attributes The clinicopathological features of CRC patients are given in Table-2. The median age of CRC patients was 59.5 years, and 64.29% were male. Most tumors (30%) were located in the sigmoid colon (Table-2). In terms of tumor grade, 33.3% of the cases were moderately differentiated, and poorly and well-differentiated cases were 23.3% and 28.57%, respectively. Most patients were in stage II (33.3%), and only 6.7% of patients showed stage IV (Table-2). Comparison of Gene Expression in Normal and Cancer Cells Significant differences were observed in the expression of MUC2, MUC5A, and MUC5B genes between normal and tumor cells so that the expression of MUC2 and MUC5A genes in normal cells was higher than in tumor cells, while tumor cells showed a higher expression of the MUC5B gene than normal cells (Figure-1). MUC5A Expression The results showed that there was a significant difference in the expression of this gene in terms of patient age (P<0.05). High levels of MUC5A gene expression were observed in patients with CRC over 50 years of age, giving an expression lower in younger patients (Figure-2A). The results showed that there was no significant difference in different tumor grades in terms of MUC5A gene expression (P=0.071). However, in poorly differentiated tumors, this gene was higher than in well-differentiated tumors (Figure-2B). The results of the present study showed that the expression of the MUC5A gene differs significantly according to the different stages of the tumor (P<0.05). The highest expression of this gene was reported in stage IV of the CRC tumor; however, no significant differences were observed between stages II and III. The lowest expression of this gene was observed in stage I of CRC (Figure-2C). There was no significant difference in the expression of the MUC5A gene in men and women with CRC (P<0.05, Figure-2D). The receiver operating characteristic (ROC) curve was used to assess the potential use of MUC5A as a predictor of CRC. The area under the curve (AUC) value for MUC5A was 0.852 (95% confidence interval [CI]=0.7181–0.9864, P=0.001, Figure-3). At the cut-off point, the sensitivity and the specificity for the MUC5A gene were 63% and 79%, respectively. MUC2 Expression In the present study, the expression of the MUC2 gene depended on patient age. There was a significant difference in the expression of this gene in CRC patients with respect to age (P=0.002). The expression of the MUC2 gene was higher in patients over 50 years of age and lower in patients less than 50 years of age (Figure-4A). The results showed that there was no significant difference in the expression of the MUC2 gene in different grades of CRC (P=0.19). However, expression of the MUC2 gene in poorly and moderately differentiated tumors was higher than well-differentiated CRC tumors (Figure-4B). CRC tumors were observed, at different stages, to express the MUC2 gene differently (P=0.021). The highest expression of the MUC2 gene was observed in stage IV CRC tumors. There was no significant difference in the expression of the MUC2 gene between stages II and III tumors, and the lowest expression of this gene was seen at stage I (Figure-4C). There was no significant difference in terms of expression of the MUC2 gene between men and women with CRC (P=0.725, Figure-4D). The AUC value for MUC2 was 0.84 at a 95% CI (95%CI=0.6963–0.9837, P=0.001, Figure-5). At the cut-off point, the sensitivity and the specificity for the MUC2 gene were 90% and 84%, respectively. MUC5B Expression The results of the current study showed that there were significant differences in the expression of the MUC5B gene according to age among patients with CRC (P<0.05). High expression of this gene has been observed in CRC patients over 50 years of age, compared to patients under fifty (Figure-6A). High expression of the MUC5B gene was observed in poorly differentiated tumors compared to moderately and well-differentiated colorectal tumors, showing a statistically significant difference (P=0.033, Figure-6B). However, there was no significant difference in the expression of the MUC5B gene in different stages of (Figure-6C), or between men and women with, CRC (Figure-6D). The AUC value for MUC5B was 0.72 (95%CI=0.547–0.893, P=0.079, Figure-7). At the cut-off point, the sensitivity and the specificity for the MUC2 gene were 85.71% and 72%, respectively.

## Discussion

In the present study, it was found that the expression of the secretory MUC genes, including MUC5A , MUC2 , and MUC5B was related to the age of the patients. The patients over 50 years of age showed high expression of these genes. The expression of the MUC5B gene was associated with different grades of CRC; however, there was no significant difference in the expression of the MUC2 and MUC5A genes. The expression of the MUC2 and MUC5A genes showed significant differences in different stages of CRC, but the expression of the MUC5B gene in different stages was not significant. There was no noticeable difference in the expression of the MUC2 , MUC5A , and MUC5B genes in terms of the gender of patients with CRC. CRC as a common, deadly, yet preventable disease has always attracted the attention of health centers around the world [22]. It is more common in men than women and increases with age so that the age of diagnosis in developed countries is about 70 years [22]. Screening has been shown to reduce the incidence and mortality rate of this cancer significantly, but there are currently no organized screening programs in most countries [22]. Over the past three decades, molecular genetics methods have been developed based on the analysis of fecal proteins, DNA, and RNA [23]. There is little information on the expression of secretory MUC genes in CRC tumors and its association with clinical variables. Therefore, the present study was undertaken to investigate the relation between the expression of secretory MUC genes and clinical variables. It has been shown that the expression of MUC2 in CRC depends on the type of tumor, indicating a decrease in the expression of this gene in colorectal adenocarcinomas [10,11] and an increase in the expression of MUC2 in mucinous carcinomas [24]. In our study, the high expression of this gene was reported in stage IV tumors compared to other stages. However, there are conflicting results in the literature regarding the expression of this secretory mucus in CRC. Some reports have suggested that the different glycosylation of MUC2 , in normal colon and in colon cancer or in relation to spliced forms of the MUC2 protein, can play contributing roles [11]. Animal studies have suggested that the inactivation of the MUC2 gene causes tumor formation in the small intestine and then in the colon [15]. In this context, the methylation of the MUC2 promoter plays an important role in reducing MUC2 expression [25]. In this study, patients over 50 years showed more expression of this secretory mucus than younger patients. This result is in line with the findings of Al-Maghrabi et al . (2019), in which high MUC2 protein expression was observed in patients over 60 years [26]. MUC2 has been shown to be a predominantly secreted mucin that is abundantly expressed in the cytoplasm of goblet cells and columnar cells [27,28]. However, there are conflicting results regarding the role of this gene in CRC, with some studies reporting downregulation of this gene during this type of cancer [21,29], while other studies have reported an upregulation [30,31]. In the current study, an increase in the expression of this gene was observed with the progression of cancer. In the present study, high expression of the MUC5A gene in stage IV as well as high expression of MUC5A , overall, was observed in patients aged over 50 years. However, there was no significant difference in different grades of CRC and gender in relation to the expression of the MUC5A gene. The role of MUC5A in different cancers varies, and it has been shown that the expression of this gene correlates with metastatic capacity in lung cancers [32], but in colon cancer, it has been stated that high expression of this gene has been associated with a better prognosis [20]. Therefore, the lack of expression of the MUC5A gene could be a prognostic factor for aggressive colorectal carcinoma. The MUC5A expression has been shown to predict a favorable outcome in CRC, and this effect is particularly strong in patients with stages II and III of the disease [33]. This was also observed in the current study. Another study found that patients with MUC5A C expressed tumors had greater overall survival [20]. In the present study, no significant differences were observed in the expression of this gene in different degrees of CRC, which is contrary to the findings of another study [34]. The present study showed that high expression of the MUC5B gene was observed in poorly differentiated colorectal tumors compared to moderately and well-differentiated tumors. The expression of the MUC5B gene has been reported to be a specific mechanism used by cancer cells to maintain a non-differentiating state [35,36]. This can be a valuable diagnostic and prognostic tool for differentiating cancer cells. In addition, abnormal expression of MUC5B has been shown in other cancers such as gastric carcinomatous tissues [37] and breast cancer tissues [38]. The current study had its own limitations. Gene expression analysis was performed solely using molecular analysis and not by immunohistochemical staining. However, the current study showed that the expression of MUC2 and the aberrant expression of MUC5A , as well as MUC5B , could be prognostic markers in CRC.

## Conclusion

Based on the results of the study, it can be stated that malignant transformation of colorectal cells was accompanied by changes in the expression of the secretory MUC genes, including MUC2 , MUC5A , and MUC5B , which can be used for diagnostic purposes. More researches are required in this regard to reach the optimal approach.

## Acknowledgment

The present study was part of a thesis written by Hossein Iranmanesh.

## Conflict of Interest

The authors declare that they have no conflict of interest.

**Table 1:** Sequences of Primers Used in the Current Study

**Genes**	**Sequences**
MUC2	F: 5’-GAGGGCAGAACCCGAAACC-3’
R: 5’- GGCGAAGTTGTAGTCGCAGAG-3’	undefined
MUC5A	F: 5’- CCATTGCTATTATGCCCTGTGT-3’
R: 5’- TGGTGGACGGACAGTCACT-3’	undefined
MUC5A	F: 5’- GCCCACATCTCCACCTATGAT-3’
R: 5’- GCAGTTCTCGTTGTCCGTCA-3’	undefined
β-actin	F: 5’- CACCATTGGCAATGAGCGGTTC-3’
R: 5’- AGGTCTTTGCGGATGTCCACGT-3’	undefined

**Table 2:** Clinicopathological Features of Colorectal Cancer Patients at Diagnosis

**Parameters**	**Values**
**Age, Median (Range)**	59.5 (32-82)
**Gender, n(%)**	undefined
Male	18 (64.29)
Female	10 (35.71)
**Tumor location, n(%)**	undefined
Cecum	4 (13.3)
Ascending colon	1(3.3)
Transverse colon	4 (13.4)
Descending colon	2 (6.7)
Sigmoid colon	9 (30)
Rectum	1 (3.3)
Rectosigmoid junction	7 (23.3)
**Grade, n (%)**	undefined
Well-differentiated	8 (28.57)
Moderately differentiated	10 (33.3)
Poorly differentiated	7 (23.3)
**Stage, n (%)**	undefined
I	5 (16.7)
II	10 (33.3)
III	8 (26.7)
V	2 (6.7)
